# Follower: A Novel Self-Deployable Action Recognition Framework

**DOI:** 10.3390/s21030950

**Published:** 2021-02-01

**Authors:** Xu Yang, Dongjingdian Liu, Jing Liu, Faren Yan, Pengpeng Chen, Qiang Niu

**Affiliations:** 1 China Mine Digitization Engineering Research Center, Ministry of Education, Xuzhou 221116, China; yang_xu@cumt.edu.cn (X.Y.); chenp@cumt.edu.cn (P.C.); 2 School of Computer Science and Technology, China University of Mining and Technology, Xuzhou 221116, China; dongjingdianliu@cumt.edu.cn (D.L.); ts18170012a31@cumt.edu.cn (J.L.); 08183005@cumt.edu.cn (F.Y.)

**Keywords:** action recognition, human pose estimation, dynamic time planning, template matching

## Abstract

Deep learning technology has improved the performance of vision-based action recognition algorithms, but such methods require a large number of labeled training datasets, resulting in weak universality. To address this issue, this paper proposes a novel self-deployable ubiquitous action recognition framework that enables a self-motivated user to bootstrap and deploy action recognition services, called FOLLOWER. Our main idea is to build a “fingerprint” library of actions based on a small number of user-defined sample action data. Then, we use the matching method to complete action recognition. The key step is how to construct a suitable “fingerprint”. Thus, a pose action normalized feature extraction method based on a three-dimensional pose sequence is designed. FOLLOWER is mainly composed of the guide process and follow the process. Guide process extracts pose action normalized feature and selects the inner class central feature to build a “fingerprint” library of actions. Follow process extracts the pose action normalized feature in the target video and uses the motion detection, action filtering, and adaptive weight offset template to identify the action in the video sequence. Finally, we collect an action video dataset with human pose annotation to research self-deployable action recognition and action recognition based on pose estimation. After experimenting on this dataset, the results show that FOLLOWER can effectively recognize the actions in the video sequence with recognition accuracy reaching 96.74%.

## 1. Introduction

Recognizing human actions can have many potential applications, including video surveillance, human–computer interfaces, sports video analysis, and video retrieval. Action recognition approaches mainly include the following three categories: based on wearable sensors [1], based on wireless signals [2], and based on vision [3,4,5]. Among them, vision-based methods have the best performance with the breakthrough of deep learning technology.

Vision-based human action recognition approach can be divided into traditional machine learning action recognition algorithm [3,6,7], deep-convolution-based action recognition algorithm [4,8,9] and human-skeleton-based action recognition algorithm [5,10,11,12,13,14]. Traditional machine learning action recognition algorithms use hand-crafted representations to extract action features. After feature normalization and feature coding, machine learning algorithms such as Support Vector Machines (SVM) [15] are used to achieve action recognition. The representative of these algorithms is iDT [7], which is widely considered as useful work. However, it cannot automatically extract features, and the computational cost of calculating optical flow is enormous [4]. Action recognition algorithms based on deep convolution features use deep convolutional networks to automatically extract the spatiotemporal information of actions in video images. According to the organization of spatiotemporal information, they can be divided into action recognition algorithms based on spatiotemporal convolution [4,16,17,18] and algorithms based on two-stream convolution networks [8,19,20]. Although these algorithms have achieved good performance in the experimental environment, they are susceptible to interference from the background environment. Simultaneously, the model needs to collect a lot of scene data for training, and the trained model cannot effectively identify new actions incrementally. Human skeleton-based action recognition algorithms usually use human pose information in the video to characterize human actions, which can effectively filter scene interference. However, due to the restriction of video-based human pose estimation algorithms [21,22,23] and the lack of video action datasets containing human pose annotations [5], previous human-skeleton-based algorithms just rely on manually annotated data [5,11] or human skeleton data obtaining from expensive motion capture equipment such as Kinects and RGB-D cameras [10], unable to effectively recognize action based on monocular video cameras.

To address these issues, we propose a novel self-deployable ubiquitous action recognition framework that enables a self-motivated user to bootstrap and deploy action recognition, called FOLLOWER. Our main idea is, based on a small number of user-defined sample action data, to establish an action fingerprint library, and then use the matching method to complete action recognition. FOLLOWER is mainly divided into guide process and follow process. The guide process uses the idea of central feature selection to build an action fingerprint library. The follow process uses an adaptive weight offset template matching to complete action recognition. However, there are some challenges in implementing this method:

- How can the standardized description of movement characteristics in different scenes and different body postures be solved?
- How can an action finger be accurately and efficiently recognized?

To address the first challenge, we design an action feature descriptor named pose action normalized feature, which can regulate different body postures based on end-to-end video 3D human pose estimation. Pose action normalized feature is mainly composed of the normalized joints series and key angle change series, which characterizes the action features of different granularities. To address the second challenge, we propose a similarity measure of action sequences called normalized joints Dynamic Time Warping (DTW)distance based on the dynamic time warping algorithm. We use it to calculate the importance of each candidate in the action class and select the most representative central pose action normalized feature as the action feature to build an action fingerprint library, with a dynamic entry mechanism to support user-defined action expansion. To address the third challenge, we use the key angle change series to quickly detect motion in video sequences and filter action to avoid unnecessary calculations. To solve the misjudgment of actions, we design an adaptive weight template matching algorithm, which can calculate the weight offset of normalized joints DTW distance for each action in action fingerprint. It can correct the similarity between the action to be recognized and actions in the action fingerprint to recognize complex types of actions available. To verify the algorithm’s effectiveness, we construct a dataset containing 14 kinds of actions for testing. Under various test scenarios, the recognition accuracy rate can reach 96.74%. Therefore, the main contributions of this article include:

- We first propose a self-deployable action recognition framework with a skeleton-based action recognition method realizing user-defined action detection and recognition under few-shot data with strong generalization ability.
- We implement action recognition based on real unlabeled video via an action feature description operator pose action normalized feature (PANF) effectively overcoming scene information interference, which improve the practical value of motion recognition algorithms based on monocular video cameras.
- We design a new template matching algorithm with low time complexity calculating offset weights to improve the effect, which can be applied to other template matching tasks.
- We construct a video action dataset containing human pose annotations, which contributes to the research on self-deployable action recognition and skeleton-based action recognition algorithms in videos. Moreover, we tested FOLLOWER on the dataset, and the recognition accuracy rate can reach 96.74%.

## 2. Framework Design

### 2.1. Overview

**Figure 1:**
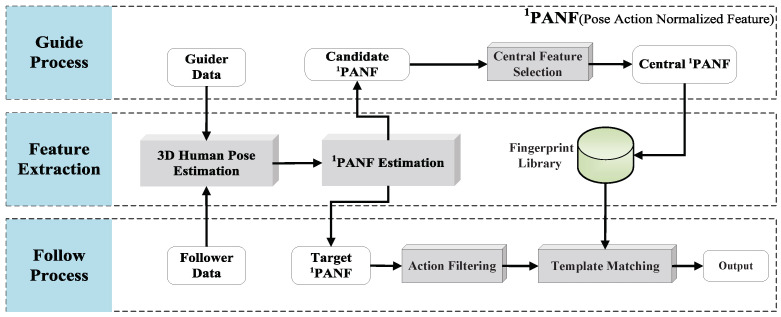
Framework architecture.

FOLLOWER is mainly divided into guide process and follow process. As shown in Figure 1, the top pipeline of the figure represents the guide process. Based on processing a small number of user-defined action video sequences with tags, FOLLOWER extracts guide pose action normalized feature and establishes an action fingerprint library based on normalized joints DTW. The action fingerprint library is not restricted by categories, which can be customized by users. The bottom pipeline of the figure shows the follow process. FOLLOWER extracts the pose action normalized feature of the real-time video sequence and matches the central pose action normalized feature of each action in the established action fingerprint library to identify the action. The middle pipeline in the figure shows the extraction process from video frames to pose action normalized features, mainly composed of the 3D human pose estimation and pose action normalized feature estimation.

### 2.2. Pose Action Normalized Feature

Pose action normalized feature is a 3D human pose-based action recognition feature description operator, which can effectively remove the scene interference to the body posture differences. Pose action normalized feature is mainly composed of the normalized joints series and key angle change series. For an action video sequence *G* of length *t*, it can be expressed as:

(1) $$G=\left\{g_{1},g_{2},\ldots ,g_{t}\right\}$$

where $g_{i}$ represents a frame containing the human body. After the 3D pose estimation, *G* obtains the 3D pose sequence *p*:

(2) $$P=\left\{\left\{p_{{}_{0,1}},p_{{}_{1,1}},\ldots p_{{}_{v-1,1}}\right\},\left\{p_{{}_{0,2}},p_{{}_{1,2}},\ldots p_{{}_{v-1,2}}\right\},\ldots ,\left\{p_{{}_{0,t}},p_{{}_{1,t}},\ldots p_{{}_{v-1,t}}\right\}\right\}$$

where $p_{i,j}$ represents the position coordinate $\left(x,y,z\right)$ of the joint point *i* in the frame *j*. *v* is the number of joint points; in this paper, v=17. After transformation, *P* can get *v* joint series $J_{i}$, which can be expressed as:

(3) $$J_{i}=\left\{p_{i,1},p_{i,2},\ldots ,p_{i,t}\right\}$$

After the pose action normalized feature estimation process, FOLLOWER can get the pose action normalized feature set *F*, which can be expressed as:

(4) $$F\left(G\right)=\left\{N\left(J\right),K\left(a\right)\right\}$$

(5) $$N\left(J\right)=\left\{Norm\left(J_{0}\right),Norm\left(J_{1}\right),\ldots ,Norm\left(J_{v-1}\right)\right\}$$

(6) $$\begin{array}{c} K\left(a\right)=\left\{\max\left(a_{0}\right)-\min\left(a_{0}\right),\max\left(a_{1}\right)-\min\left(a_{1}\right),\ldots ,\max\left(a_{k-1}\right)-\min\left(a_{k-1}\right)\right\} \end{array}$$

Pose action normalized feature set *F* is mainly composed of the normalized joints series *N* and key angle change series *K*, used to build action fingerprint library and match actions. *K* represents the fine-grained features of actions in time series and *N* represents the global coarse-grained features of actions. Function Norm is the normalized change of the joint and *a* is a set of key angle sequence.The length of *a* is *k*, and each angle in $a_{i}$ is calculated by corresponding 3D pose $P_{i}$ in *P*.

#### 2.2.1. 3D Human Pose Estimation

To guarantee performance and efficiency, 3D human pose estimation is composed of the human detector Yolo-v3 [24], 2D human pose estimation Pruned HRnet, and 2D to 3D pose estimation Video Pose 3D [25]. Pruned HRnet is a lightweight model we designed to improve the real-time performance of the algorithm, which is obtained based on HRnet using the channel pruning method with self-determined pruning parameters. Yolo-v3 and Video Pose 3D use pre-training model directly.

**Figure 2:**
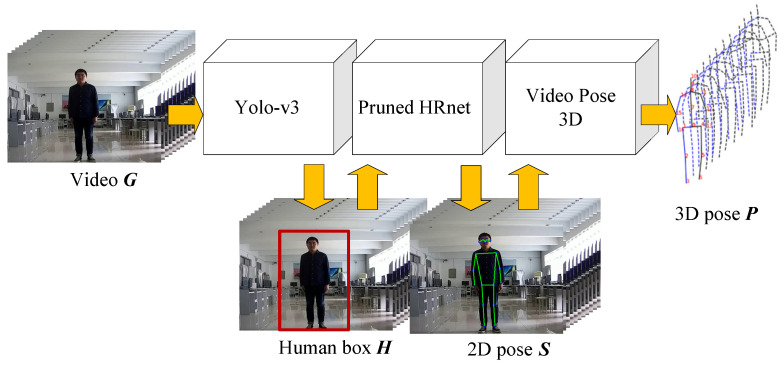
3D human pose estimation.

As shown in Figure 2, we utilize a video action sequence *G* to generate 3D pose series *P*. Yolo-v3 generates the detection box *H* of the human body. *H* generates 2D human pose *S* through Prund HRnet. VideoPose3D performs 3D pose estimation through adjacent multi-frame sequences, and finally obtains 3D pose series *P*.

To lighten the algorithm, we prun the original HRnet model. In the original HRnet [26], to achieve reliable high-resolution representations, the algorithm connects multiple high-resolution subnetworks in parallel. It performs various multi-scale fusions, which leads to a complicated model structure, and effective pruning is complicated.

**Figure 3:**
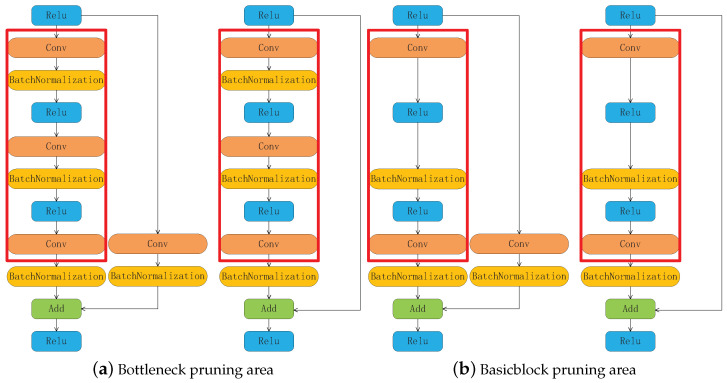
HRnet basic network structure pruning area.

The essential components of HRnet can be divided into BasicBlock, Bottleneck, and Multi-scale Fusions layer. The multi-scale fusion layer contains more information and fewer parameters, so it is not pruned during the pruning process. The structures of BasicBlock and Bottleneck are shown in Figure 3, both of which are residual structures. The convolution operation before the Add operation does not involve pruning because it involves multi-scale fusions. The rest of the layers include pruning, and the pruning area is shown in the red box in Figure 3.

The pruning strategy draws on the network slimming algorithm [27]. The batch-normalization layer (BN layer) coefficient γ is directly used as the criterion for measuring the importance of the channel. The function of the BN layer is generally expressed as:

(7) $$y=\gamma \times \frac{x-\mathrm{Mean}\left(x\right)}{\sqrt{\mathrm{Var}\left(x\right)}+\varepsilon }+\beta$$

Each channel *C* corresponds to a set of γ and β. γ represents scale parameters, β represents shift parameter, and ε represents normalization parameter. The higher the value of γ is, the more important *C* is. Given a pruning rate 999,999,999, each γ in the BN layer that needs to be pruned is collected and sorted to obtain the sequence *L*, whose length is *m*. The global threshold is $\theta_{g}$:

(8) $$\theta_{g}=L_{m\;\times \;p}$$

For a BN layer to be pruned, the γ sequence is *B*, and its local threshold of γ is $\theta_{local}$:

(9) $$\theta_{local}=\left\{\begin{array}{cc} \max\left(B\right) & \max\left(B\right)\leq \theta_{g}\\ \theta_{g} & \max\left(B\right)>\theta_{g} \end{array}\right.$$

Within a BN layer, *C* has a γ that is smaller than $\theta_{local}$, which is the target of pruning.

The pruning rate λ is selected by plotting the scatterplot of the pruning rate λ and the performance indexes acc and ap of the model after pruning on the COCOVal2017 data set [28], making sure to maximize pruning rate λ with particular acc and ap.

We sample 50 candidates λ interval in the interval $\left[0,1\right]$ and obtain 50 candidates Pruned HRnet by pruning. After testing, we obtain the corresponding acc and ap, and the corresponding set is $L_{acc}$ and $L_{ap}$. Let the abscissa be *x*, and the ordinate is *y*:

(10) x=λ

(11) $$y=\frac{acc}{\max\left(L_{acc}\right)}+\frac{ap}{\max\left(L_{ap}\right)}$$

By drawing a scatterplot of *y* versus *x*, we find that the distribution of scatters is in the form of a convex function, so the model corresponding to the inflection point is selected as Prund HRnet. Simultaneously, according to the size of the model, we conducted pruning training with a higher pruning rate and achieved a higher degree of model compression.

**Figure 4:**
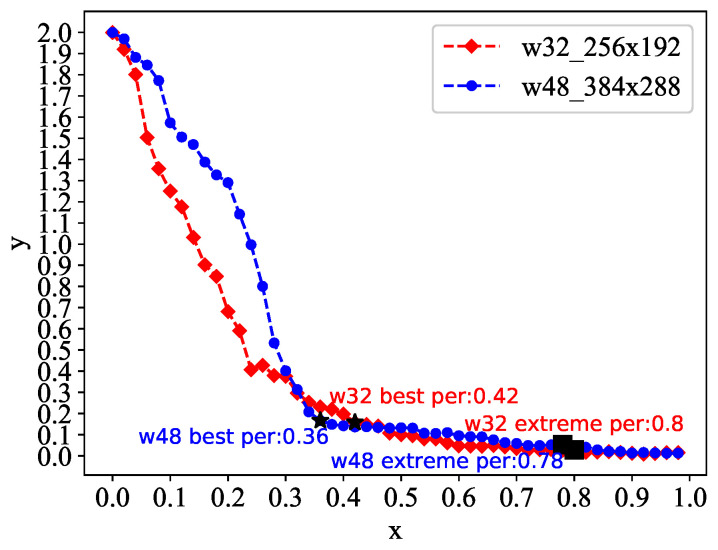
Candidate model selection.

We select w32-256x192-HRnet and w48-384x288-HRnet in the original model and sample 50 candidate pruning rates 999,999,999 equally in the interval $\left[0,1\right]$ to prune the models. Candidate models are quickly tested on the validation set to obtain the corresponding performance indicators of acc and ap. The scatter plot is shown in Figure 4.

We selected four candidate models for training: w48-best, w48-extreme, w32-best, and w32-extreme. After training, we have a model that balances accuracy and computational complexity as the 2D pose estimation model.

#### 2.2.2. Pose Action Normalized Feature Estimation

Pose action normalized feature estimation mainly includes coordinate transform, scale transform, and key angle calculation. Among them, coordinate transform and scale transform correspond to the function Norm.

**Figure 5:**
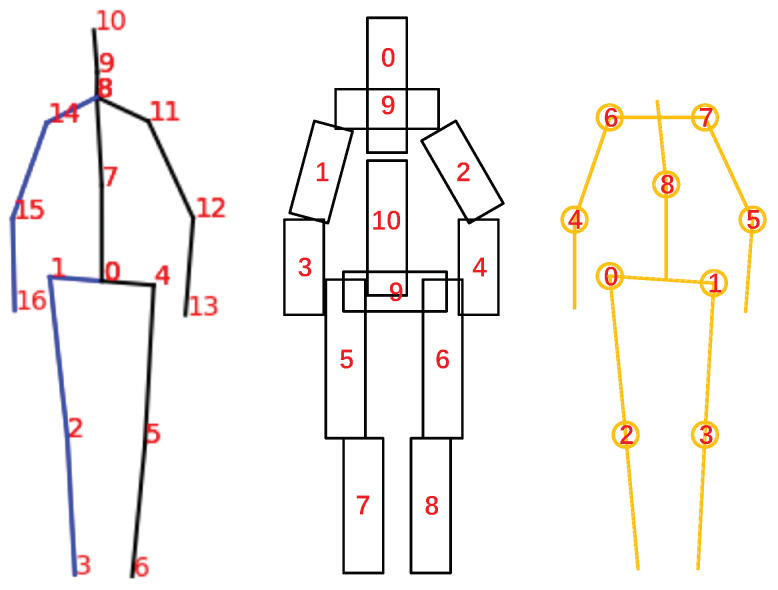
Human pose information.

The visual representation of the 3D pose is shown in Figure 5. There are 17 joints in one pose.

The coordinates of each joint generated by 3D human pose estimation are absolute coordinates relative to $H_{i}$. As the $H_{i}$ coordinate system changes, the value of the joint coordinates also changes. Therefore, coordinate transform is needed to obtain coordinate descriptions that are not related to $H_{i}$. In FOLLOWER, the revised human body central point is selected as the coordinate origin:

(12) $$O_{ti}=\mathrm{Mid}\left(\mathrm{Mid}\left(\mathrm{Mid}\left(J_{1,ti},J_{4,ti}\right),\mathrm{Mid}\left(J_{11,ti},J_{14,ti}\right)\right),J_{7,ti}\right)$$

Mid is the midpoint calculation function.

Affected by human body shape and shooting location, the human body posture generated by 3D human body posture estimation will have great differences in scale. Therefore, this paper designs a human body scale description method based on the human body scale. As shown in Figure 5, the pose is divided into 11 blocks with the staff *r*:

(13) $$r=\sum_{i=0}^{e-1}\mathrm{Dist}\left(J_{S_{i},1},J_{E_{i},1}\right)/11$$

*S* and *E* represent the coordinate pair set of connection relationship between the joints, whose length is *e*. The line between $S_{i}$ and $E_{i}$ represents a set of skeletons. From this, we can get the normalized function of the joint:

(14) $$\begin{array}{c} \mathrm{Norm}\left(J_{i}\right)=\left\{\left(J_{i,1}-O_{1}\right)/r,\left(J_{i,2}-O_{2}\right)/r,\ldots ,\left(J_{i,t}-O_{t}\right)/r\right\} \end{array}$$

At the same time, we consider that some key angle information can also describe human limb movements. Based on this, as shown in Figure 5, we selected nine key angles as features to represent the changes in the progress of the legs, arms, and torso, which is used for motion threshold analysis and action filtering.

### 2.3. Guide Process

In the guide process, for a new action, the algorithm firstly extracts the pose action normalized feature corresponding to each guider in the standard guider action set *G* to construct the candidate pose action normalized feature set *C*. Then, according to the similarity between action sequences based on normalized joints DTW distance, we select the center feature in the action class to obtain the most representative center pose action normalized feature as the feature of the action. Finally, the algorithm stores the center pose action normalized feature in the action fingerprint library for the follow process.

#### 2.3.1. Normalized Joints DTW

For FOLLOWER, calculating the similarity measure between two action video sequences $G_{1}$ and $G_{2}$ is the core of establishing an action fingerprint library and matching actions. We designed the DTW distance based on the normalized joint sequence set in pose action normalized feature, normalized joints DTW, as the index of the similarity measure.

Let the normalized joints series of $G_{1}$ and $G_{2}$ be $N_{1}$ and $N_{2}$, respectively, which are both composed of *v* normalized joint series. Since the normalized joint series is a time series of coordinate position information, the DTW algorithm is used to calculate the similarity of two-time sequences, which can be unequal in length. The calculation formula of DTW distance is shown in the literature [29].

Considering the interference of noise in practical applications, before performing the DTW calculation, the original $N_{1}$ and $N_{2}$ need to be equally downsampled to obtain the time series $D_{1}$ and $D_{2}$ with the equally divided sampling parameter sp. sp is the length of each group of joint series in $D_{1}$ and $D_{2}$. Calculating the average DTW of joint series in *v* groups $D_{1}$ and $D_{2}$, we calculate normalized joints DTW distance as follows:

(15) $$\mathrm{NJDTW}\left(F_{1},F_{2}\right)=\sum_{i=0}^{v-1}\mathrm{DTW}\left(D{}_{1},D{}_{2}\right)/v$$

$F_{1}$ and $F_{2}$ represent the pose action normalized feature of $G_{1}$ and $G_{2}$. For the two action video sequences $G_{1}$ and $G_{2}$, the smaller is the normalized joints DTW distance, the higher is the degree of similarity.

#### 2.3.2. Action Fingerprint Library

Our identification method is to match actions in the action fingerprint library. Suppose there are *n* kinds of actions, each of which has *m* guiders. If candidate pose action normalized features are directly stored as features in the action fingerprint library for matching, space and time complexity is at least $O\left(mn\right)$. Besides, if there are noises in some guider actions during recording, the accuracy of action recognition will also be affected. Therefore, we propose a method for selecting the center feature in the action class, which dynamically selects the most representative guider pose action normalized feature in each action as the central pose action normalized feature of this class when a new guider action is recorded in the fingerprint library. The action fingerprint library only stores central pose action normalized feature of each class, so matching complexity is $O\left(n\right)$.

For the candidate pose action normalized feature set of action *C*, whose length is *m*, the sum of the normalized joints DTW distance of each candidate $C_{i}$ and another candidate pose action normalized feature $C_{j}$ is used as the criterion for measuring the importance of $C_{j}$. The smaller is the value, the more likely the candidate pose action normalized feature is to obtain a lower normalized joints DTW distance than another candidate pose action normalized features. The candidate pose action normalized feature with minimum of normalized joints DTW distance is selected as the representative of this action. Thus, we can get the selection function of the central feature:

(16) $$\min_{i}\;\sum_{j=0}^{m-1}\mathrm{NJDTW}\left(C_{i},C_{j}\right)$$

The obtained $C_{i}$ is stored in the action fingerprint library as the central feature of the action.

To reduce the entry time of the fingerprint library, the action fingerprint library stores the list of the sum of the normalized joints DTW distance of each $C_{i}$ and other $C_{j}$, which is represented by *U*, and the index corresponding to the center pose action normalized feature $c_{i}$. When recording a new candidate pose action normalized feature $C_{m}$, the update algorithm for $c_{i}$ and *U* is as follows:

(17) $$c_{i}=\left\{\begin{array}{cc} c_{i} & U_{c_{i}}+\mathrm{NJDTW}\left(C_{m},C_{c_{i}}\right)\leq \sum_{j=0}^{m-1}\mathrm{NJDTW}\left(C_{m},C_{j}\right)\\ m & U_{c_{i}}+\mathrm{NJDTW}\left(C_{m},C_{c_{i}}\right)>\sum_{j=0}^{m-1}\mathrm{NJDTW}\left(C_{m},C_{j}\right) \end{array}\right.$$

(18) $$U_{j}=\left\{\begin{array}{cc} U_{j}+\mathrm{NJDTW}\left(C_{m},C_{j}\right) & j<m\\ \sum_{j=0}^{m-1}\mathrm{NJDTW}\left(C_{m},C_{j}\right) & j=m \end{array}\right.$$

The algorithm’s time complexity is $O\left(m\right)$, which is convenient for users to add guider-data by themselves.

### 2.4. Follow Process

In the follow process, FOLLOWER continuously captures the video sequence of vt frames as the sequence to be recognized. It takes the latest pt frame as a priori video for motion detection based on the key angle change series threshold. If a motion is detected, the system takes the captured video sequence as follower data and extracts its pose action normalized feature as *X* to represent the pose action normalized feature to be recognized, whose length is at least vt frames. Through the action filtering process based on key angle changes and the adaptive weight offset template matching process based on normalized joints DTW distance, FOLLOWER selects the class with the smallest distance from *X* as the predicted class within the action fingerprint library.

#### 2.4.1. Motion Detection

Before action recognition, the algorithm needs to determine whether the currently acquired video frame sequence contains motion. Let the previous pt frame of the series be the priority video $V_{pr}$, calculate the corresponding key angle change sequence $K_{pr}$, and take the difference between the maximum angle change and the minimum angle change as the motion estimation value:

(19) $$prv=\max\left(K_{pr}\right)-\min\left(K_{pr}\right)$$

The higher is the value of prv, the greater is the amplitude of motion. Let the motion threshold be prp. When the value of prv is lower than the motion threshold prp, the algorithm considers that the motion has not started, and thus does not perform action recognition. When the value of prv exceeds the motion threshold prp, the algorithm begins to capture the sequence of video frames for recognition and recognizes the sequence of video frames after the end of the motion. The judgment for the end of the motion is to perform motion detection after intercepting the vt frames until the value of prv is lower than the motion threshold prp. $K_{pr}$ can be calculated by Equation (6).

#### 2.4.2. Action Filtering

Before the action matching, the key angle change series information of the action in the fingerprint library is compared with the key angle change series information of the follower action, which can filter out some actions with significant differences on the global level. The algorithm first sorts key angle change series in descending order and obtains the corresponding subscript sequence $S_{a}$. $S_{a}$ reflects the arrangement of the changing intensity of the key angle. For different actions, the order of $S_{a}$ arrangement is different. Considering that, in actual applications, different users have a certain difference in the overall understanding of actions, filtering directly with $S_{a}$ is likely to lead to misjudgment; thus, we design the filtering parameter fp. If they are inconsistent, the algorithm considers that follower does not belong to the category corresponding to guider and skips the category directly without performing action matching. The filtering parameter fp of different actions is different, requiring the user to adjust according to the specific action. Actions that are easily misjudged, the value of fp is higher.

#### 2.4.3. Adaptive Weight Offset Template Matching

Action fingerprint library can get candidate action set for action matching after action-filtering, let their pose action normalized feature set as *Y*. We count the normalized joints DTW distance between *X* and each pose action normalized feature $Y_{i}$ in set *Y*. The action corresponding to the subscript *i* of the minimum value is the prediction of the follower data. In the actual test, we find that, being affected by factors such as motion complexity, the normalized joints DTW distance calculated by different actions are not evenly distributed. To describe this difference, we design the function Mc, which is the average of the normalized joints DTW distance between the center pose action normalized feature $A_{i}$ of each class and all center pose action normalized feature $A_{j}$ in the action fingerprint library:

(20) $$\mathrm{Mc}\left(i\right)=\sum_{j=0}^{n-1}\mathrm{NJDTW}\left(A_{i},A_{j}\right)/n$$

The smaller is the Mc value of a class, the more likely is the normalized joints DTW distance that can be calculated by this class with other sequences to take a smaller value, thus different sequences have a higher probability of being judged as this class.

Based on this, we modify the template matching algorithm and design an adaptive bias weighting function ABW based on Mc values to solve the difference in class probability distributions caused by differences in Mc values. ABW is an exponential function with a base less than 1, which decreases monotonically according to the value of Mc. Let Mcmean be the average value of Mc values of all classes, maxv be the maximum value of Mc after normalization, and its corresponding weight offset be maxp, expressed as follows:

(21) $$Mcmean=\sum_{i=0}^{n-1}\mathrm{Mc}\left(i\right)/n$$

(22) $$maxv=\max\left(Mcl\right)/Mcmean$$

(23) $$maxp=\frac{vw}{maxv}$$

where vw is the parameter to adjust the function ABW and Mcl is the list of all Mc values. maxv and maxp can determine the base of ABW function, expv:

(24) $$expv=maxp^{\frac{1}{maxv}}$$

From this, the expression of the ABW function can be obtained as:

(25) $$\mathrm{ABW}\left(i\right)=expv^{\frac{\mathrm{Mc}\left(i\right)}{Mcmean}}$$

Let the similarity between *X* and each pose action normalized feature $Y_{i}$ in *Y* be $\mathrm{ABWDTW}\left(i\right)$:

(26) $$\mathrm{ABWDTW}\left(i\right)=\mathrm{ABW}\left(i\right)\times \mathrm{NJDTW}\left(X,Y_{i}\right)$$

The subscript of the category label corresponding to the video sequence to be recognized is pi:

(27) $$\min_{pi}\;\left(\mathrm{ABWDTW}\left(pi\right)\right)$$

To reduce the start-up time of real-time recognition, the algorithm performs Mc calculation in advance after the generation of the action fingerprint library and saves the corresponding weight in a local file, which can be used directly in action matching.

### 2.5. Framework Parameters

There are six user-defined parameters in FOLLOWER: number of sampled frames pt and motion threshold prp in motion detection, sampling parameters sp in NJDTW calculation, number of follower action recognition sampling frames vt, filtering parameters in action filtering fp, and adjustment parameter vw of function ABW. Users can directly use the default values we provide or adjust these parameters to adapt to different application scenarios. The specific information is as follows.

pt is adjusted according to the sampling frequency of the video. In general, the pt value increases as the sampling frequency of the video increases, which can capture significant motion.

prp affects the sensitivity of motion detection. The smaller is the prp, the easier it is to detect motion. The appropriate value of prp is adjusted according to the motion estimation value prv of the fixed sequence and the motion sequence.

sp represents the granularity of the action, whose value affects the grain size of recognition in action matching. If the sp value is too large, the probability of noise will increase, and, if it is too small, the characteristics of the action will be lost.

vt represents the length of the action. The larger is the vt, the longer is the duration of the action that can be recognized. When adjusting the vt parameter, the value of sp should be considered. If there is too much difference between them, the action feature will also be lost. vt is related to the video’s frame rate of the video; the higher is the frame rate, the higher is the value of vt that should be selected.

fp is related to the complexity of the movement and degree of pose change. Actions with a smaller degree of posture change and more elaborate action steps are easily misjudged, whose corresponding fp value may be higher.

vw is used to adjust the ABW function, which can adjust the weight offset. The lower is the weight of vw, the higher is the degree of bias, which is suitable for the action feature library with a significant difference in Mc value. When vw is equal to maxv, the value of the function ABW is fixed at one, which means that the adaptive weight offset template matching downgrade to conventional template matching algorithm.

## 3. Dataset

**Figure 6:**
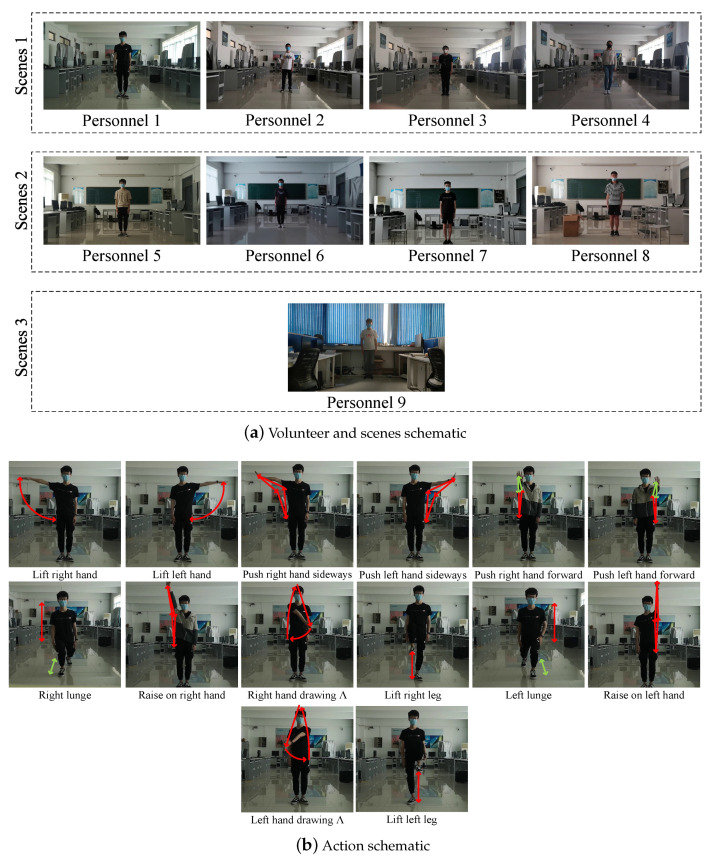
Dataset schematic.

We collected single person action video datasets containing 14 kinds of actions for nine persons with different body types, which can be used for temporal action localization. As shown in Figure 6, the experiment devices are ordinary smartphones. In a single video, an action is continuously performed more than 10 times. The duration of each action is between 2 and 3 s, and there is a 1–3 s interval between each action. The dataset information is shown in Table 1.

The dataset mainly includes three scenes. The scenes of Volunteers 1–4 are the same, but their postures are quite different. The scenes of the Volunteers 5–8 are the same, but the object positions of the scene are changed during the acquisition process. The scene of Volunteers 9 is quite different from other volunteers.

We manually cropped the first four actions of each video to create a video action classification dataset, which is used to test the action classification algorithm and build the action fingerprint library.

Based on the 3D human pose estimation algorithm in the method, we simultaneously extracted the 2D human pose and 3D human pose from all videos constructing a video-based action pose dataset that can be used for action recognition research based on pose estimation.

## 4. Experiment

We evaluated FOLLOWER on the dataset we collected with different parameters and guider data to showcase: **(i)** effectiveness of the functional component designed in FOLLOWER; **(ii)** accurateness of FOLLOWER in different action fingerprint library; and **(iii)** analysis of critical factors affecting the algorithm.

### 4.1. Implementation Details

**Environment.** In these experiments, we implemented our FOLLOWER in a massive batch of experiments with different action fingerprint library construction methods. We conducted experiments on a personal computer, with the Intel Core i7 8th Gen as CPU and the NVIDIA GTX 1070 as GPU. The deep learning framework was PyTorch with CUDA10.1 and Cudnn7.

**Parameter.** Since the pace of actions of each person was not consistent during data collection, there is an absolute difference in the number of continuous frames of actions. We adjusted the parameter vt of different action video to reduce the misidentification of actions caused by action positioning errors, as shown in Table 2.

To prevent the action from being erroneously filtered out during the action filtering process, the fp value of each action is not less than 8, as shown in Table 3.

The other value in the following experiments are fixed: sp=30, prp=2.5, and pt=6.

vw is the core parameter of the action classification algorithm, whose value was obtained in the following experiments.

**Evaluation Metrics**. We used error recognition rate and recognition accuracy to evaluate our algorithm. The error recognition rate is the sum of the error detection rate and the error classification rate. The error detection rate is generated by the error segmentation of the action caused by the motion detection algorithm. The error classification rate is the ratio of an incorrectly identified number to the total number. Recognition accuracy is the ratio of a correctly identified number to the total number. Therefore, the sum of error detection rate and detection accuracy may not be 1, as error segmentation may lead to more recognized actions than the number of actual actions.

### 4.2. Effectiveness of Functional Component

We intercepted the first action sequence in each video of Volunteers 1–4 as guide data to build action fingerprint library to test the effectiveness of the functional component designed in FOLLOWER.

**Purned HRnet.** We retrained four candidate models selected based on Figure 4 on the COCO2017 dataset, which is used to learn 2D pose estimation. The training epoch is the same as the initial epochs of 210 rounds. The comparison results are shown in Table 4.

Params and GFLOPs represent the computational cost of the model, where smaller values are better. To ensure the accuracy and computational cost of the model, we finally chose the w48-extreme model as the 2D pose estimation model, for which the Params compression ratio is 70.44% and the GFLOPs compression ratio is 70.21% with higher acc and ap than the original HRNet-W32.

**Figure 7:**
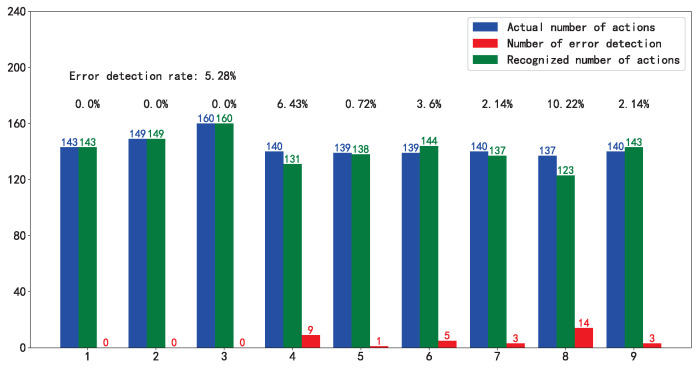
Motion detection.

**Motion Detection.** Influenced by personnel understanding of action and frequency of movement, we tested motion detection for each person separately. Error detection rate was used to assess the effect. As shown in Figure 7, the overall error detection rate is 5.21% under the control of default parameters. Motion detection algorithm can capture motion.

**Action Filtering**. After segmenting the video action segment, we tested action filtering in units of actions. The measured index was the matching speedup ratio ar, which is calculated from the number of filtered actions nf divided by the number of action matches without filtering na:

(28) ar=na/nf

The test results are shown in Table 5. The overall matching speedup ratio is 1.76, and the acceleration ratio of each action is not less than 1.4. It means that the action filtering algorithm can effectively improve the speed of recognition.

**Figure 8:**
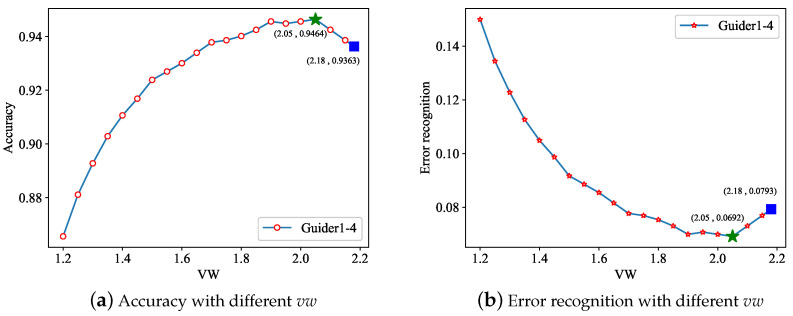
Template matching with different vw.

**Adaptive Weight Offset Template Matching.** Different from regular template matching, we used vw to adjust the matching value. To prove the effectiveness of adaptive weight offset template matching, we sampled vw every 0.5 in the interval of $\left[1.2,maxv\right]$ to test the follow process and determine the best value of vw. The results are shown in Figure 8. The star mark corresponds to the best performance of adaptive weight offset template matching, and the square corresponds to the performance of regular template matching. We can find that the performance of the adaptive weight offset template matching with suitable vw is better than regular template matching.

### 4.3. Comparison Test with Different Action Fingerprint Library

To thoroughly test the performance of the algorithm, we used multiple sets of guiders to build action fingerprint library to test our algorithm. Unlike deep learning methods, our “train data” are far fewer than the test data. For each action class in action fingerprint library, we only used four segmented guider data to test nearly 90 action samples. The performance is shown in Table 6.

**Comparison with different scenes.** We built action fingerprint libraries based on similar scenes, and each scene contains information about multiple people. Experiment Guiders 1–4 correspond to Scene 1 with different postures and experiment Guiders 5–8 correspond to Scene 2 with some scene interference. The recognition results of both are above 92%. Although the accuracy is lower than the optimal value of its containing personnel single-person fingerprint library, recognition effect is more stable than single datum. The result of a database containing multiple postures is better than that of multiple scenarios, which means that we can focus on collecting multi-posture data in practical applications.

**Figure 9:**
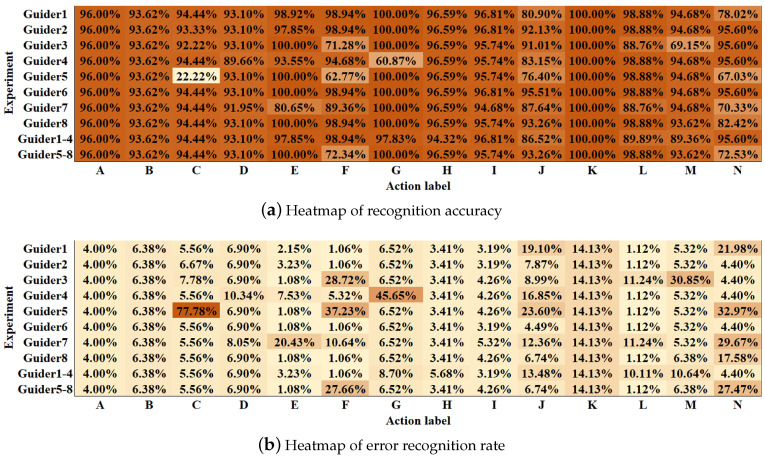
Heatmap of performance.

**Comparison with different personalities.** We separately used the data of Volunteers 1–8 to build action fingerprint libraries, each of which involves only one person data. As the shown by the results for experiment Guiders 1–8, the recognition accuracy can reach up to 96.74%. Nevertheless, there is a problem of unstable recognition. In particular, the recognition effect of the fingerprint library based on the action of Volunteer 5 is inferior, only 85.63%. We checked the data of Volunteer 5 and found that there are missing frames and picture damage, which affect the final recognition result. Combined with Figure 9, we can easily find the action classes that affect the recognition results. It can be found that the problems that affect the stability of recognition are mainly concentrated on these actions, which can guide us to correct the fingerprint database by replacing the guider data of these actions.

### 4.4. Ablation Study

To further understand the influence of each component on the recognition result, we conducted ablation experiments based on the fingerprint library of Guiders 1–4. First, we manually segmented the first four actions of each action video of Volunteers 1–8 to test the recognition effect of action filtering and matching without detection. Then, we analyzed the effects of filtering and matching with detection. Finally, to analyze the impact of scene interference, we only tested the data corresponding to Scene 1.

As shown in Table 7, we can find that detection limits the display of recognition accuracy. The addition of filtering can filter out some actions that affect the recognition result, effectively improving the recognition effect. Besides, in the scene comparison test, the recognition effect is not much different, which further illustrates the recognition stability of our algorithm.

## 5. Potential Applications

To better demonstrate our framework, we show the FOLLOWER’s practical value through a usage example in this section. FOLLOWER can be applied to the field of smart home remote control, where users can bind control instructions and custom operations to achieve user-defined control. Compared with the traditional approach, FOLLOWER only requires a monocular camera with low deployment cost.

First, users can make arbitrary actions as guidance data. FOLLOWER only needs to collect 3–4 action datasets to complete the action fingerprint library data preparation. Then, FOLLOWER extracts the pose information of the mentor data and stores it in the pose database to construct the action fingerprint library.

In the actual work, the user only needs to perform corresponding actions in front of the camera. The FOLLOWER automatically recognizes the user action information and then triggers the corresponding control signal to achieve intelligent control of the furniture. FOLLOWER supports the action recognition of different users in different scenarios, so users can share the control experience by sharing the action fingerprint library.

## 6. Conclusions

This paper presents the design of a unified description method of human actions in video sequences based on 3D pose estimation. Combining dynamic time warping, motion detection, action filtering, and adaptive weight offset template matching, we bring about incremental motion detection and recognition under a small amount of data guidance and parameter control. Adaptive weight offset template matching can be applied to other template matching application scenarios. At the same time, we propose a single-person action recognition dataset containing 3D pose markers, which contributes to the development of action recognition research based on pose estimation.

## Figures and Tables

**Table 1:** Dataset information.

Label	Action	Quantity
A	Lift right hand	100
B	Lift left hand	94
C	Push right hand sideways	90
D	Push left hand sideways	87
E	Push right hand forward	93
F	Push left hand forward	94
G	Right lunge	92
H	Raise on right hand	88
I	Right hand drawing “⋀”	94
J	Lift right	89
K	Left lunge	92
L	Raise on left hand	89
M	Left hand drawing “⋀”	94
N	Lift left leg	91

**Table 2:** Parameter vt of action.

Volunteer Number	Action Label	vt
1	A∼K, M∼N	65
	L	70
2	A∼F, H∼L, N	45
	G	70
	M	65
3	A∼N	80
4	A∼N	70
5	A∼N	70
6	A∼N	70
7	A∼N	70
8	A∼N	70
9	A∼N	65

**Table 3:** Parameter fp of actions

Label	Action	fp
A	Lift right hand	8
B	Lift left hand	8
C	Push right hand sideways	8
D	Push left hand sideways	8
E	Push right hand forward	8
F	Push left hand forward	8
G	Right lunge	8
H	Raise on right hand	8
I	Right hand drawing ⋀	9
J	Lift right leg	9
K	Left lunge	8
L	Raise on left hand	8
M	Left hand drawing ⋀	9
N	Lift left leg	9

**Table 4:** COCOVal2017 dataset test results.

Model	Scale	Params	GFLOPs	acc	ap	$ap^{50}$	$ap^{75}$	ar
HRNet-W32	256×192	28.5M	7.1	0.883	0.765	0.935	0.837	0.841
HRNet-W48	384×288	63.6M	32.9	0.887	0.781	0.936	0.849	0.86
w32-best	256×192	17.9M	4.4	0.882	0.763	0.936	0.837	0.841
w48-best	384×288	43.8M	21	0.888	0.781	0.936	0.849	0.859
w32-extreme	256×192	7.5M	2.2	0.863	0.732	0.926	0.813	0.809
w48-extreme	384×288	18.8M	9.8	0.885	0.775	0.935	0.847	0.853

**Table 5:** Action filtering acceleration effect.

Label	na	nf	ar
A	1344	864	1.56
B	1246	799	1.56
C	1190	595	2
D	1162	585	1.99
E	1316	658	2
F	1302	651	2
G	1372	883	1.55
H	1190	595	2
I	1274	639	1.99
J	1190	804	1.48
K	1470	945	1.56
L	1232	616	2
M	1246	651	1.91
N	1218	794	1.53
Overall	17,752	10,079	1.76

**Table 6:** Performance with different action fingerprint library.

Experiment	maxv	vw	Guider	Follower	Error Recognition	Accuracy
Guider1	1.69	1.5	1	1–9	7.15%	94.41%
Guider 2	2.11	1.6	2	1–9	5.28%	96.27%
Guider 3	1.85	1.85	3	1–9	9.95%	91.61%
Guider 4	2.22	2	4	1–9	9.56%	92.00%
Guider 5	3.95	3.4	5	1–9	15.93%	85.63%
Guider 6	2.22	2.15	6	1–9	4.82%	96.74%
Guider 7	1.59	1.25	7	1–9	10.18%	91.38%
Guider 8	3.46	1.4	8	1–9	6.06%	95.49%
Guiders 1–4	2.18	2.05	1–4	1–9	6.92%	94.63%
Guiders 5–8	3.78	3.3	5–8	1–9	8.70%	92.85%

**Table 7:** Ablation test.

Detection	Filtering	Matching	Guider	Follower	Error Detection	Accuracy
×	✓	✓	1–4	1–8	2.01%	97.99%
✓	×	✓	1–4	1–9	8.08%	93.47%
✓	×	×	1–4	1–9	5.21%	94.79%
✓	✓	✓	1–4	1–4	5.91%	95.27%
✓	✓	✓	1–4	1–9	6.92%	94.63%
